# Non-Small Cell Lung Cancer With Synchronous Peritoneal Adenocarcinoma: A Rare Independent Combination

**DOI:** 10.7759/cureus.10166

**Published:** 2020-08-31

**Authors:** Shobha Mandal, Ravi R Pradhan, Mary Grace Bethala, Salman Khan, Apurwa Karki

**Affiliations:** 1 Internal Medicine, Guthrie Robert Packer Hospital, Sayre, USA; 2 Internal Medicine, Tribhuvan University Institute of Medicine, Kathmandu, NPL; 3 Internal Medicine, GlobeHealer, Philadelphia, USA; 4 Pulmonary and Critical Care Medicine, Geisinger Medical Center, Danville, USA; 5 Critical Care, Guthrie Robert Packer Hospital, Sayre, USA

**Keywords:** squamous cell carcinoma, lung, peritoneal carcinomatosis, independent tumors

## Abstract

Lung cancer is one of the most common malignancies worldwide, and metastasis occurs in more than one-third of cases. Common sites of metastatic disease are the brain, spine, nerve, adrenal glands, bone, liver, and pleura. Peritoneal involvement, however, is rare, and peritoneal involvement found in lung cancer is presumed to be metastatic until proven otherwise. This is due to the fact that primary peritoneal adenocarcinoma is uncommon and difficult to distinguish from the metastatic spread.

Here, we report on a case of a 73-year-old woman who presented with ascites. Evaluation of ascitic fluid was consistent with adenocarcinoma. Subsequent CT of the chest revealed a 4.3-cm mass in the lower lobe of the left lung, which was determined to be poorly differentiated squamous cell carcinoma on histopathology. This is a rare case of two synchronous primary cancers: adenocarcinoma and poorly differentiated squamous cell carcinoma.

To conclude, physicians should be familiar with an independent presentation of squamous cell carcinoma of the lung and peritoneal carcinomatosis in the same patient, as the outcome of independent tumors is poor in most cases.

## Introduction

Lung cancer is the leading cause of cancer deaths worldwide in men and is the second most common in women [1]. Risk factors for lung cancer are cigarette smoking (95% in men and 90% in women in the United States), radon, asbestos, arsenic exposure, secondhand smoke, and other chemicals [2]. It is classified as non-small cell (NSCLC, 85%) and small cell lung cancers (SCLC, 15%). NSCLC is further classified as adenocarcinoma (38.5% of all lung cancers), squamous cell carcinoma (20%), and large cell carcinoma (3%) [3]. The five-year survival rate after the diagnosis of lung cancer is 15.6%, which is less than the survival rate of breast, colon, or prostate cancer [2]. The prognosis of SCLC is worse compared to NSCLC because of its aggressive nature. The most common cause of cancer-related death in lung cancer patients is the distal metastasis of the tumor, and nearly 40%-60% of cases of lung cancer exhibit distal metastasis at the time of diagnosis [4]. The sites of metastasis are organs like the brain, spine, nerve, adrenal gland, bone, liver, pleura, and rarely the peritoneum [5]. 

The peritoneum is a two-layered continuous membrane covering the abdominal and pelvic cavities. Peritoneal carcinomatosis (PC) is the metastasis of primary cancer to the peritoneum, and it is a less common site of metastasis compared to other sites. The term PC was first coined in 1931 by Sampson for metastatic involvement of the peritoneal stromal surface by ovarian cancer cells [6]. The most common cancers involving the peritoneum are gastrointestinal (GI), reproductive, genitourinary tracts, and less commonly pancreas, appendix, prostate, and lung [7]. Only 5% of lung cancers metastasize to the peritoneum. Any lung cancer involving the peritoneum has a poor prognosis with overall survival of fewer than two months [8]. 

The majority of patients with lung cancer present in advanced stages involving multiple organs, including peritoneum. Lung cancer can metastasize to the peritoneum and has a poor prognosis and survival [8]. Peritoneal cancer can have independent primary other than lung cancer; hence, all peritoneal cancers need further investigation to rule out any other independent cancers like in our patient. Initially, our patient was thought to have advanced lung cancer with metastasis to the peritoneum; however, on biopsy, two primary synchronous malignancies were discovered. She was diagnosed with adenocarcinoma of peritoneum and squamous cell carcinoma of the lung. 

## Case presentation

**Figure 1:**
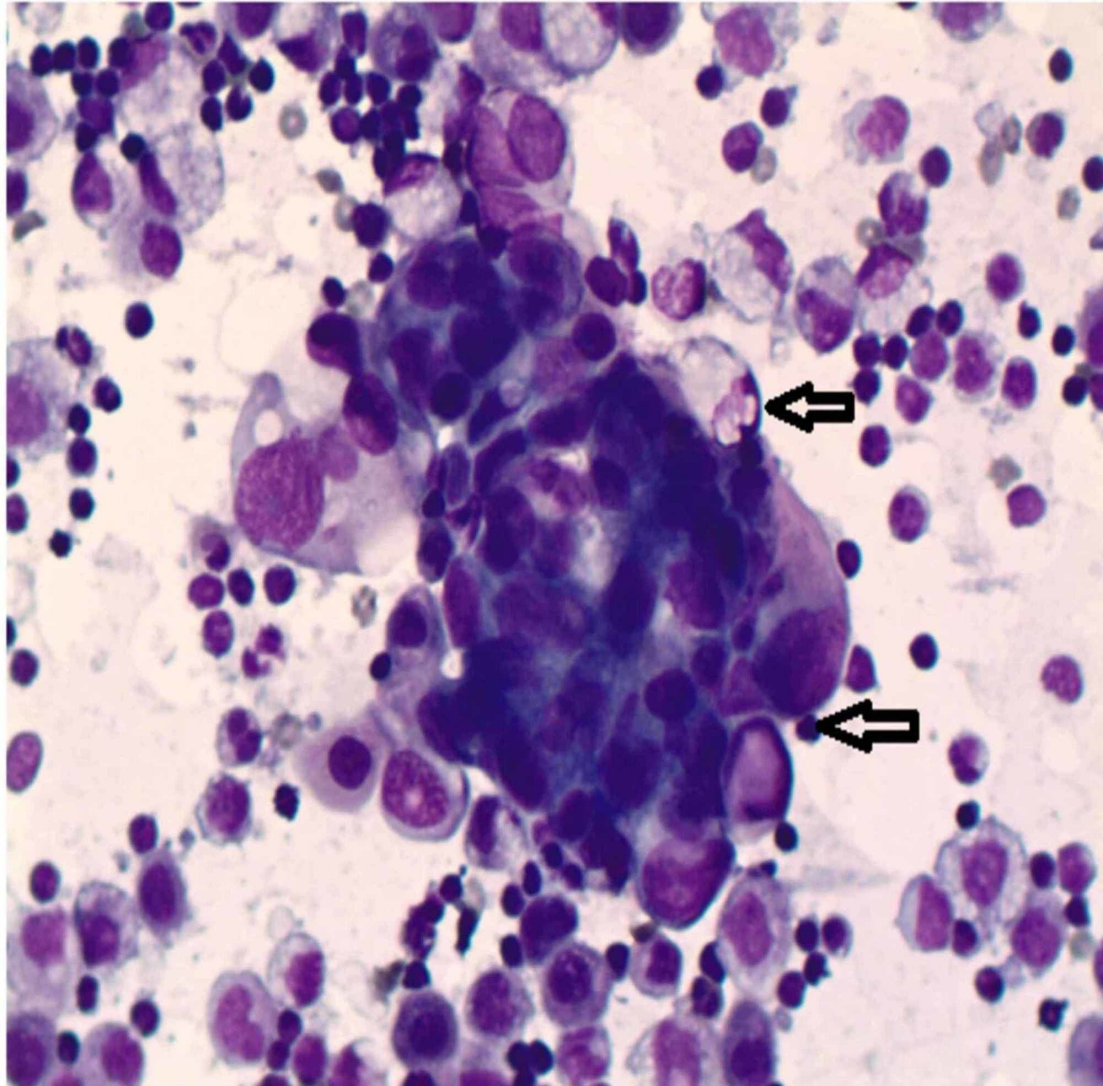
Ascitic fluid cytology finding consistent with adenocarcinoma shown by arrows (Diff-Quick stain, ×400 magnification)

A 73-year-old previously healthy female who was a heavy smoker (60 pack-years) presented to our center with chief complaints of insidious onset and gradually progressive abdominal distension, acid reflux, bloating, and loss of appetite for the last six months. She tried over-the-counter antacids with minimal relief. On examination, the patient had a temperature of 37°C, heart rate of 70 beats per minute, blood pressure was 120/75 mm Hg, respiratory rate of 20 breaths per minute, and oxygen saturation of 96% on room air. Examination of the abdomen revealed gross ascites. On chest examination, there was decreased breath sounds bilateral. The remainder of the physical examination was unremarkable. She underwent paracentesis with the removal of 3.5 liters of ascitic fluid. The analysis of ascitic fluid was consistent with adenocarcinoma (Figure 1).

CT scan of the abdomen and pelvis revealed abdominopelvic collections and features suggestive of PC. 

**Figure 2:**
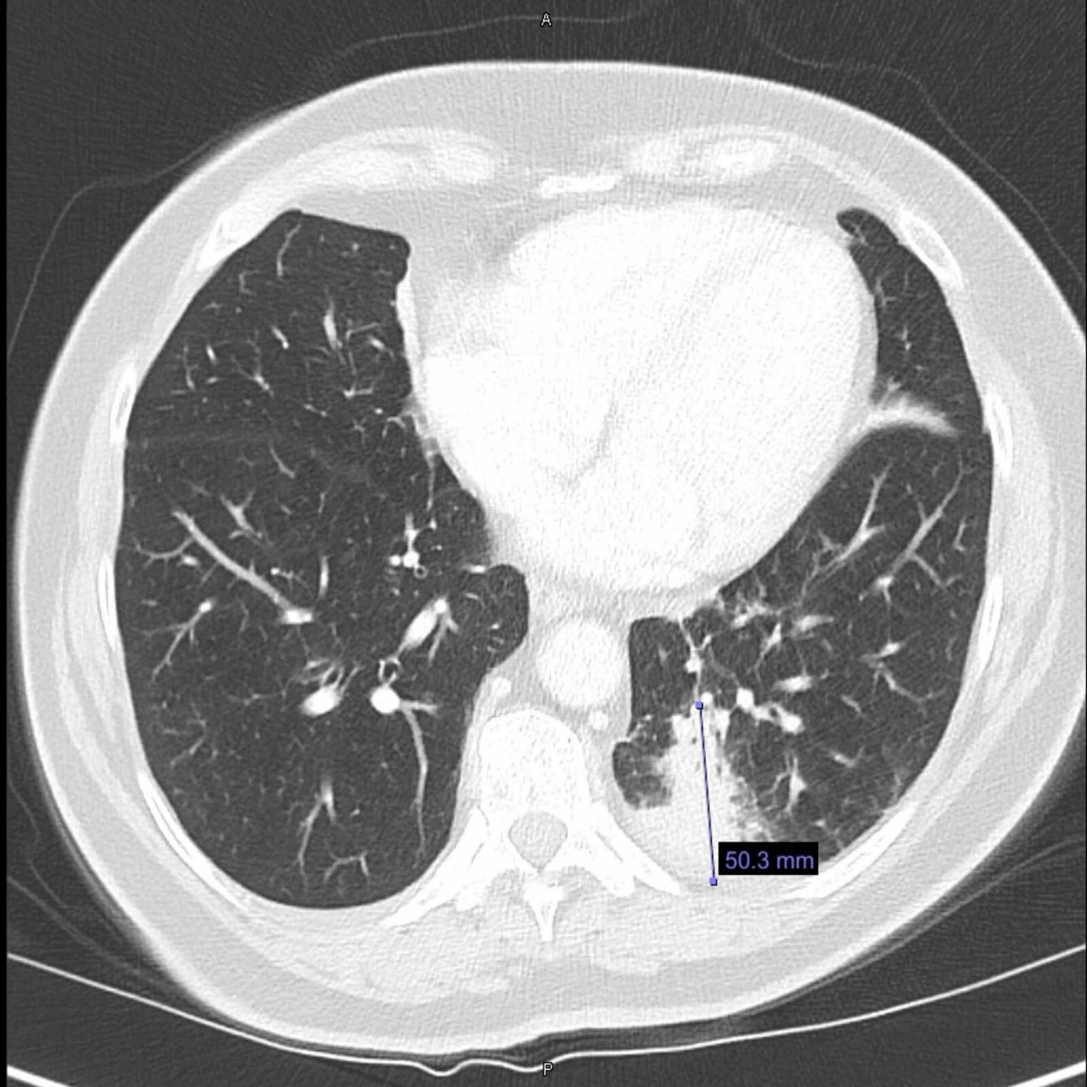
A CT scan of the chest further shows a 50.3-mm mass in the lower lobe of the left lung along with an enlarged left-sided mediastinal lymph node.

A CT scan of the chest further showed a 50.3-mm mass in the lower lobe of the left lung along with an enlarged left-sided mediastinal lymph node (Figure 2).

**Figure 3:**
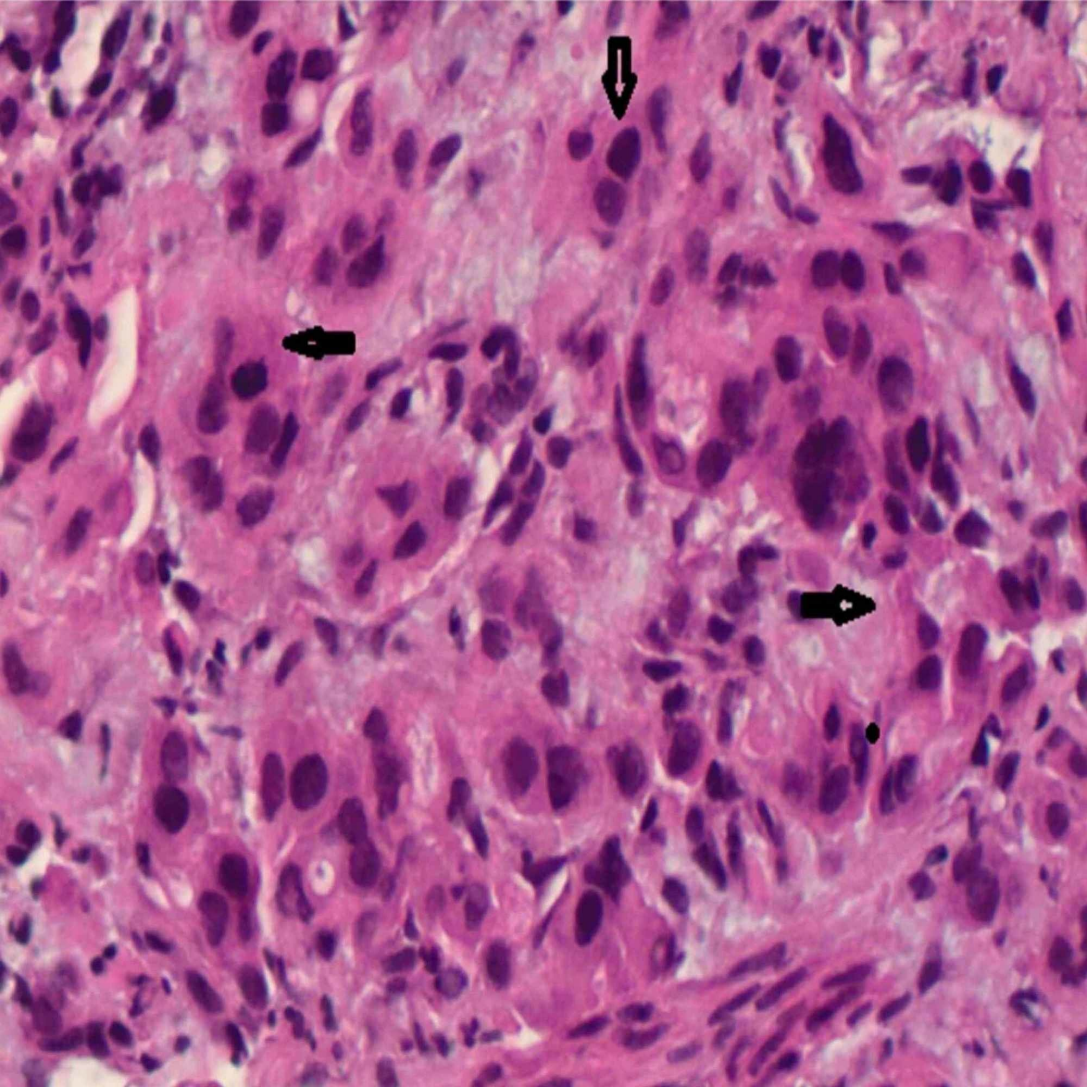
Bronchoscopy with endobronchial ultrasound-guided biopsy of the lung mass consistent with poorly differentiated non-small cell carcinoma of lung, shown by arrows (Hand-E, ×400 magnification)

Bronchoscopy with endobronchial ultrasound-guided biopsy of the lung mass consistent with poorly differentiated non-small cell carcinoma of the lung (Figure 3).

**Figure 4:**
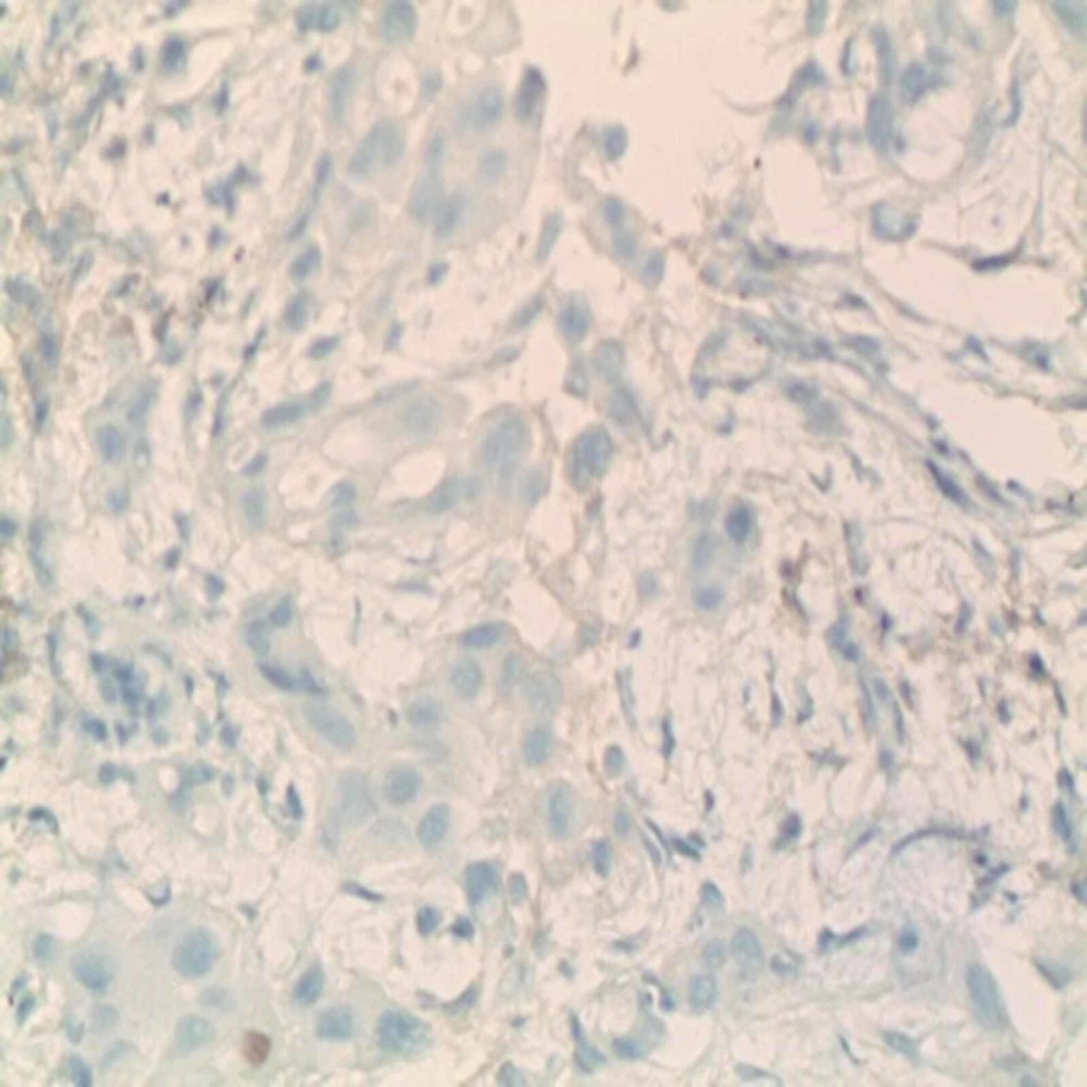
Immunohistochemistry showing tumor cells negative for P40 (magnification ×400)

**Figure 5:**
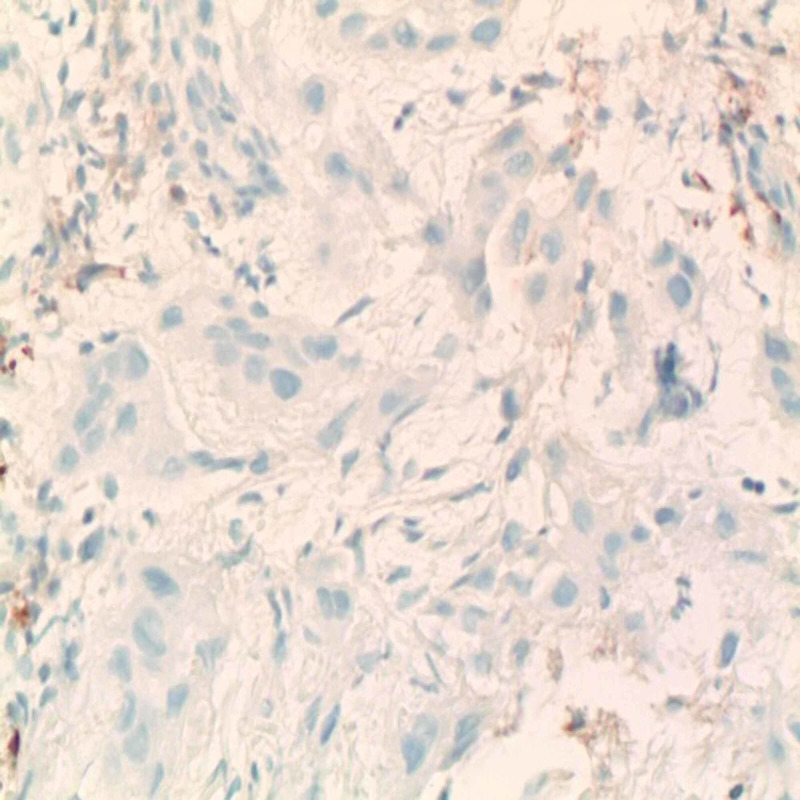
Immunohistochemistry showing tumor cell negative for TTF1 (magnification ×400)

The immunohistochemistry was positive for CK7 and CK5/6 and negative for P40 (Figure 4) and TTF1 (Figure 5).

 

**Figure 6:**
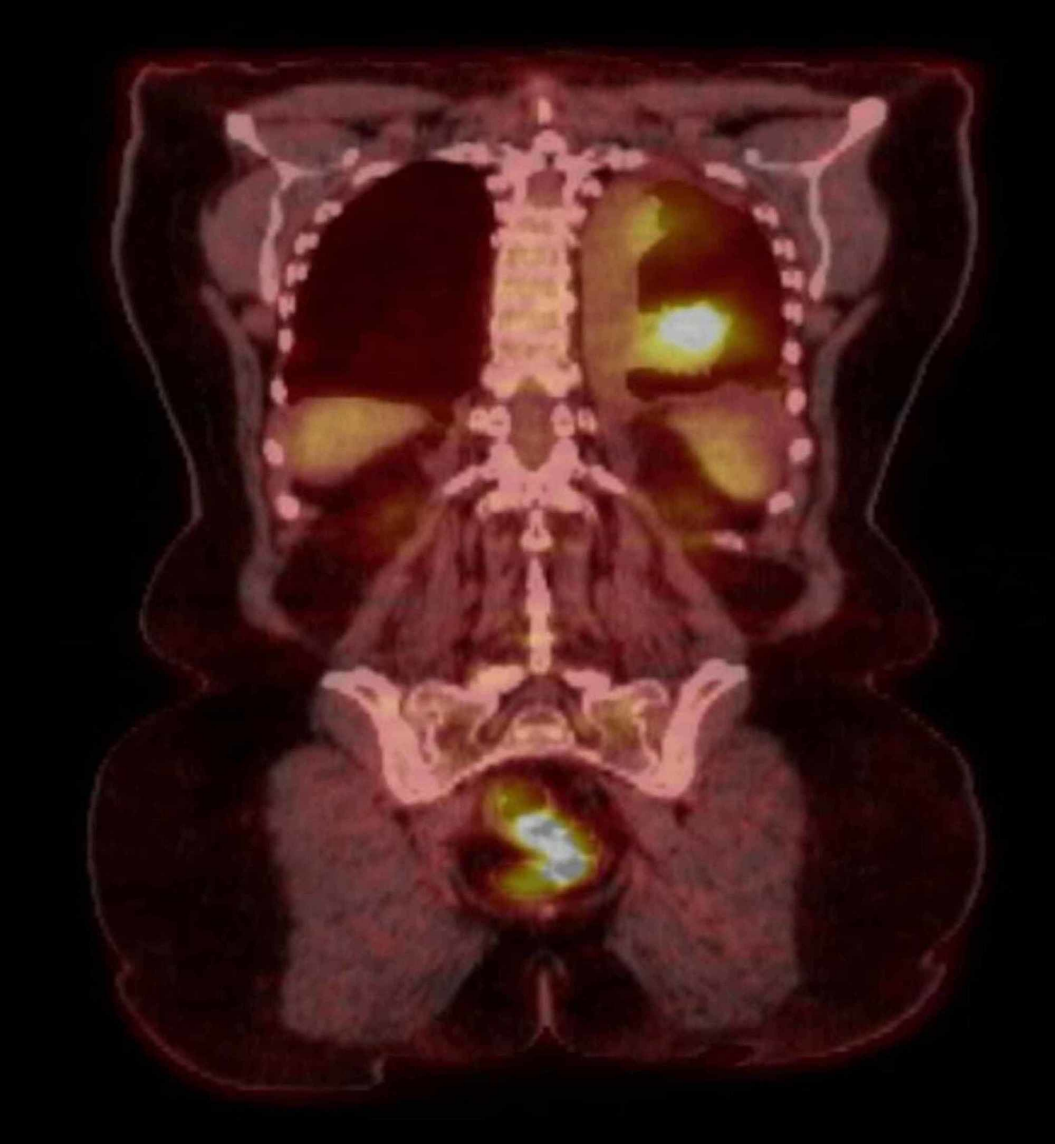
Positron emission tomography (PET) scan shows increased uptake in the left lower lobe of the lung and hilar and subcarinal lymph nodes

Napsin, CK20, PAX8, calretinin, WT1, CDX2, GATA3, and GCDFP15 were suggestive of a poorly differentiated squamous cell carcinoma. MRI of the brain was negative for any metastasis. Positron emission tomography (PET) scan showed increased uptake in the left lower lobe of the lung and hilar and subcarinal lymph nodes (Figure 6).

Pelvic ultrasound showed a large bulky uterus secondary to multiple intramural fibroids with thickening of the endometrium. The patient had no vaginal bleeding. Further workup showed carcinoembryonic antigen (CEA) 1.2 (normal <3 ng/ml) and alpha-fetoprotein (AFP) 1.6 (normal <10 ng/dl). She was offered an endometrial biopsy, but she refused. 

Given her squamous cell carcinoma of the lung, she was planned to be started on platinum-based doublet chemotherapy (carboplatin and paclitaxel) along with an immune checkpoint inhibitor (pembrolizumab) every three weeks for four cycles, followed by maintenance therapy with pembrolizumab every three weeks. She reported improvement in abdominal distension and bloating with weekly therapeutic paracentesis, but dyspnea continued. She was started on for three liters of oxygen via the nasal cannula. 

After her initial diagnosis, the patient began to have progressive weakness and was unable to walk without support. The goals of care were discussed, and the patient elected to not continue with aggressive cytotoxic chemotherapy. She was started on hospice care after a detailed discussion of treatment and prognosis. Unfortunately, she passed away a few months later. 

## Discussion

Most patients with lung cancer are diagnosed in the advanced stage with metastasis to different parts of the body. In recent studies, overall survival for these patients was found to be very poor with maximum survival of nine months despite aggressive treatment [7]. 

Peritoneal carcinoma can be asymptomatic in its early stage but can be incidentally discovered during radiological imaging, diagnostic testing, or surgical exploration for unrelated reasons. Once symptomatic, patients may present with abdominal pain, discomfort, bloating, and feelings of fullness. They can also present with ascites and bowel obstruction on physical examination, although this is seen in less than 50% of patients [7]. 

Among all peritoneal cancer, only 3% have a primary peritoneal origin, with the remainder secondary to metastasis from other primary cancers. Of those cases for which PC is metastatic, the primary malignancy is most commonly ovarian cancer (46%) [9], followed by breast cancer (41%), lung cancer (21%), malignant melanoma (9%), colorectal cancer (7%) [10], extra-abdominal malignancy (10%), and around 3%-5% are of unknown origin [10]. It is seen more commonly in women, among which 70% are elderly [11]. It is difficult to diagnose PC based on clinical presentation; hence, further workup with ultrasonography, CT, and MRI scan of chest, abdomen, and pelvis, 18F-fluorodeoxyglucose (FDG) PET and biopsy of the tumor can be considered for confirmation of the type of cancer cell. Recently, a scoring system proposed by Dr. Sugarbaker named peritoneal carcinomatosis index (PCI) has been used for the patient selection for surgery, prognosis, and outcome [12]. 

However, whenever there is a finding suggesting the possibility of peritoneal involvement, clinicians should keep a low threshold for investigating underlying primary cancer and should always consider biopsy and ascitic fluid analysis of both malignancies to differentiate primary and metastatic peritoneal cancer, as in our case. Our patient presented with the complaint of abdominal bloating as an initial symptom despite the presence of her advanced-stage lung cancer. In our case, the patient was found to have adenocarcinoma on cytologic analysis of ascitic fluid analysis; a CT scan of the abdomen demonstrated lymph node involvement in the left lung. Initially, it was assumed to be a metastasis from the lung to the peritoneum, but later biopsy of the lung tissue came positive for independent NSCLC. Like in our case, a patient presenting with peritoneal involvement and lung or other cancer is assumed to have peritoneal metastasis. Still, it can be two different malignancies occurring together, and it can be missed if proper investigations with biopsies are not done to confirm. Therefore, in our case, we are presenting two independent cancers diagnosed together, which could have been easily missed assuming it to be the metastasis from the lung. In our patient, we were not able to figure out whether the peritoneal cancer was primary cancer or spread from adjacent organs as the patient refused the endometrial biopsy and another further workup. 

PC can be managed by complete cytoreductive surgery combined with hyperthermic intraperitoneal chemotherapy and systemic chemotherapy. So far, this treatment has been used for the management of tumors of the GI, genitourinary tract, appendiceal, colorectal, gastric, ovarian, and neuroendocrine tumors. Despite the use of these treatment modalities, the overall prognosis of tumors involving the peritoneal cancer is very poor, and studies have shown the overall survival to be nine months the maximum [13]. Hence, a multidisciplinary team approach, including palliative care, is needed for improved patient care and outcomes. 

## Conclusions

In our patient, the peritoneal adenocarcinoma was possibly secondary to metastasis from underlying endometrial malignancy, as the patient had thickened endometrium. Unfortunately, the patient declined tissue biopsy of the endometrium, so we were unable to confirm the origin of the peritoneal adenocarcinoma. Our patient had poorly differentiated stage IV NSCLC with simultaneous peritoneal adenocarcinoma, representing two synchronous primary malignancies. The management of two separate malignancies requires highly individualized treatment plans that are likely dissimilar to standard practices in the setting of a single tumor. Consideration must be given to the chemotherapy regimen that improves symptoms and prolongs survival while taking the goals of therapy into account.
